# Prevalence and Clinical Severity of Erectile Dysfunction in Patients Undergoing Coronary Angiography: A Descriptive Cross-Sectional Study at a Tertiary Hospital in Northern Sri Lanka

**DOI:** 10.7759/cureus.82125

**Published:** 2025-04-12

**Authors:** Thurairajasingam Vipusuthan, Kumanan Thirunavukarasu, Guruparan Mahesan, Rajeshkannan Nadarajah

**Affiliations:** 1 Internal Medicine, Teaching Hospital Jaffna, Jaffna, LKA; 2 Medicine, Faculty of Medicine, University of Jaffna, Jaffna, LKA; 3 Cardiology, Teaching Hospital Jaffna, Jaffna, LKA; 4 Family Medicine, Wentworthville Medical and Dental Centre, Sydney, AUS

**Keywords:** coronary artery disease, erectile dysfunction, ischemic heart disease, vasculogenic risk factors, international index of erectile function

## Abstract

Background: Erectile dysfunction (ED) is an early clinical manifestation, and it could also be the "tip of the iceberg" of chronic atherosclerotic disease. As the onset of ED precedes major cardiovascular events, screening for it could be a simple and cost-effective approach for preventive management in low-income countries like Sri Lanka. This study aimed to determine the prevalence and clinical severity of ED among male patients undergoing coronary angiography, to assess the association between ED severity and the extent of coronary artery disease (CAD), and to examine relevant clinical risk factors.

Methods: A descriptive cross-sectional study was conducted among male patients undergoing coronary angiography at Teaching Hospital Jaffna, Sri Lanka, from July 2024 to October 2024. All eligible patients were recruited, which represents 73.9% (298) of the estimated sample. A structured interviewer-administered form was utilized to collect the data, which included a validated International Index of Erectile Function (IIEF)-5 questionnaire for assessing ED. Coronary angiography findings were used to assess the severity of CAD. IBM SPSS Statistics for Windows, Version 29 (Released 2021; IBM Corp., Armonk, New York, United States) was used to analyze the data.

Results: Out of 298 male patients who underwent coronary angiography during the study period, 181 were found to have ED (60.7%; CI: 55.1-66.2). There was a statistically significant association between ED severity and CAD extent (p-value < 0.001), particularly notable in patients with multi-vessel disease. The proportion of patients with severe ED (IIEF index = 4) showed an increased number of affected coronary vessels. Among patients with triple vessel disease, 82.5% (52 out of 63) had severe ED, while in patients with single-vessel disease and normal epicardial coronary vessels, severe ED was much less prevalent (5.7% and 4.9%, respectively) (Spearman-correlation r = 0.637, p-value < 0.001). The presence of hypertension was significantly associated with ED (p-value = 0.026) as well as the duration of hypertension (p-value = 0.034) in bivariate analysis. After adjustment for potential confounders, the results showed that diagnosed CAD was associated with a 3.92 times (CI: 2.2-7.03) higher risk of having ED.

Conclusion: This study demonstrated a strong association between ED and CAD, and it highlights that ED is an early indicator of underlying CAD. Routine screening for ED in high-risk patients, particularly those with hypertension and diabetes, can improve early detection of CAD and aid in the implementation of preventive strategies. However, the study's limitations, including its single-center design and cross-sectional nature, highlight the need for further multi-center and longitudinal research to validate these findings and explore the potential therapeutic benefits of ED management in cardiovascular disease.

## Introduction

Erectile dysfunction (ED) is a common problem in male sexual health, especially among men with underlying vascular disease. ED was defined by the National Institute of Health in 1993 as the inability to attain sustainable erections required for successful intercourse [1]. Addressing its pathogenesis can be complex due to its multifactorial background. Penile erections are produced by an integration of physiologic processes involving the hormonal, vascular, and central and peripheral nervous systems. The most important cause for ED is vascular pathology (endothelial dysfunction) [2]. The pathophysiological mechanism for ED, which is the early impairment of vasodilatation and later obstructive atherosclerotic plaques in penile blood vessels, shares a similar pathological basis for the development of coronary artery disease (CAD) [3]. Atherosclerosis, which affects arterial blood flow, is a common cause of arterial impotence. The pathogenesis of ischemic cardiovascular diseases is also largely dependent upon this crucial mechanism, which explains why men with ischemic heart disease (IHD) often report ED [4]. Both ED and CAD share common risk factors, including hypertension, insulin resistance, dyslipidemia, obesity, inactivity, positive familial history of CAD, smoking, and depression, as indicated by the Massachusetts Male Aging Study [5]. As both ED and IHD share similar pathophysiological mechanisms and risk factors, they can be considered as different presentations of a single disease. The drugs used in CAD have complex effects on erectile function, with diuretics and beta-blockers having unfavorable effects. The outcome would be poor drug compliance and a lack of clinic follow-up [6].

Even though ED and CAD share common pathophysiological processes and similar risk factors, only a limited number of studies have been conducted in Sri Lanka due to the sensitive nature of the topic and associated social stigma. The symptoms of ED can develop before the symptoms and signs of cardiovascular disease. In contrast to the larger coronary, internal carotid, and femoral arteries, the penis has smaller vessels that could be affected by plaque burden more rapidly. Therefore, ED is an early indicator of future cardiovascular disease events, making it a crucial finding to take primary and secondary preventive measures [7]. The evaluation of ED is a straightforward and inexpensive prognostic tool that is a substitute for expensive investigations and can guide early, conclusive investigations and management. It cuts down time, money, and human resources. This study aimed with a primary objective of determining the prevalence and clinical severity of ED among male patients undergoing coronary angiography, with the secondary objective to assess the association between ED severity and the extent of CAD and to examine relevant clinical risk factors.

## Materials and methods

This cross-sectional study was conducted in the cardiology wards and coronary care unit (CCU) of the Teaching Hospital Jaffna, Jaffna, Sri Lanka, for a period of four months between July 2024 and October 2024. The Ethical Review Committee of the Postgraduate Institute of Medicine granted approval under reference number ERC/PGIM/2024/061. The study population consisted of male patients who were aged between 18 and 60 and undergoing coronary angiography, either in the cardiology ward or the CCU during the study period. Patients with mental incompetence to provide consent and patients with spinal cord or brain injuries, Parkinson's disease, stroke, radiotherapy to the prostate, and local penile disease such as Peyronie’s disease and cavernous fibrosis who underwent urological surgeries were excluded. The sample size was calculated with the following formula:

$n = \frac{Z^2 \cdot P \cdot (1 - P)}{e^2}$

where n is the sample size, Z represents the confidence level at 95% (1.96), P denotes the prevalence, and e is the margin of error.

As no studies have been done in Sri Lanka among patients with CAD regarding the prevalence of ED, the estimated prevalence (P) was taken as 50%. The margin of error (e) was set at 5%, and the non-responders' rate was assumed to be 5%; hence, the required sample size was 403. All the patients who met the inclusion criteria during the study period were recruited consecutively (consecutive sampling) from all eligible male patients aged 18-60 years undergoing coronary angiography during the four-month study period.

We were able to recruit 298 (73.9% of the estimated sample size of 403) due to limited eligible patients, availability of angiography, time constraints, practical challenges, and the study being conducted at a single tertiary hospital. A structured (interviewer-administered) data collection form was utilized to gather the data. It consists of four components. Parts A, B, and C relate to candidate demographic data, comorbidities, medications such as beta blockers and thiazides, and the result of the coronary angiogram, respectively. These data were obtained from both the patient's bed head ticket (BHT) and clinic books by the investigator. Part D includes a validated translation of the International Index of Erectile Function (IIEF)-5 questionnaire in the patient's native language, which was employed to assess ED. This was a self-administered component that was given to the patient as a separate form, which was filled out by the patient after giving informed consent. The IIEF questionnaire used to evaluate ED was already validated, and a translated version of the IIEF questionnaire used by an earlier researcher who studied the prevalence of ED among the diabetic population in Sri Lanka was utilized after gaining permission [8,9]. ED was classified into the following five severity levels: scores 22-25 indicated none, 17-21 indicated mild, 12-16 indicated mild-moderate, 8-11 indicated moderate, and 1-7 indicated severe. This index was used for both descriptive and inferential analysis.

While other tools such as the penile Doppler ultrasonography, nocturnal penile tumescence (NPT) testing, or neurovascular assessments exist, these are either expensive or technically complex and not routinely available in public hospital settings in low-income countries like Sri Lanka and not validated locally. As such, the validated IIEF questionnaire was used to assess ED.

CAD diagnosis criteria

CAD was diagnosed based on visual estimation of ≥70% stenosis in at least one major epicardial coronary artery, performed by an experienced interventional cardiologist. The term "significant narrowing" refers to a diameter stenosis of over 70% in any major epicardial vessel in the "worst" angiographic view. Depending on the number of vessels affected, patients are categorized as having single-vessel, double-vessel, or triple vessel disease [10].

Statistics and data analysis

Data were analyzed by using IBM SPSS Statistics for Windows, Version 29 (Released 2021; IBM Corp., Armonk, New York, United States). Categorical variables were presented as values and percentages. Continuous variables were presented as mean ± standard deviation or median ± IQR. The chi-square test/Fisher’s Exact Test and Spearman Correlation tests were used to analyze associations or correlations between categorical data. Spearman's rank correlation was used for correlation, if appropriate. Tables and graphs were used to represent data. A p-value less than 0.05 was considered statistically significant. Logistic regression (backward stepwise) analysis was done by using variables that showed an association with ED in bivariate analysis (age, education, hypertension, diabetes mellitus, CAD). However, smoking status was not entered in the model, which showed a negative association for ED in bivariate analysis, and it is not explainable by the pathogenesis of ED (model summary: -2 log likelihood = 328.63, Cox & Snell R Square = 0.211, Nagelkerke R Square = 0.286).

## Results

The mean age of 298 men who underwent coronary angiography during the study period was 52.8 + 6.43, with a minimum age of 30 and a maximum age of 60. The majority of them completed secondary education (174, 58.4%); 114 (38.2%) completed primary education only, and 10 (3.4%) completed tertiary education.

Out of 298 male patients who underwent coronary angiography, ED prevalence was found to be 60.7% (181 out of 298 patients, CI: 55.1-66.2). Furthermore, 70% (152 out of 217 patients) of patients underwent coronary angiography and were also diagnosed with CAD. Further analysis showed a significant association between ED severity and extent of CAD (χ² = 273.73, p-value < 0.001). Notably, the prevalence of severe ED was significantly higher in patients with triple vessel disease (52 out of 63, 82.5%) than in those with normal coronary vessels (4 out of 81, 4.9%) (Table 1).

**Table 1:** Severity of ED based on the IIEF index with the number of coronary vessels involved CAD: coronary artery disease; ED: erectile dysfunction; IIEF: International Index of Erectile Function

Coronary angiogram findings of studied cases		IIEF index - ED severity					Total case for CAD severity
		0	1	2	3	4	
		No ED	Mild ED	Mild to moderate ED	Moderate ED	Severe ED	
0	Normal coronary vessel	52 (64.2%)	14 (17.3%)	8 (9.9%)	3 (3.7%)	4 (4.9%)	81 (100.0%)
1	Single-vessel disease	47 (53.4%)	5 (5.7%)	28 (31.8%)	3 (3.4%)	5 (5.7%)	88 (100.0%)
2	Double vessel disease	14 (21.2%)	4 (6.1%)	6 (9.1%)	33 (50.0%)	9 (13.6%)	66 (100.0%)
3	Triple vessel disease	4 (6.3%)	2 (3.2%)	1 (1.6%)	4 (6.3%)	52 (82.5%)	63 (100.0%)
Total cases ED severity group		117 (39.3%)	25 (8.4%)	43 (14.4%)	43 (14.4%)	70 (23.5%)	298 (100.0%)
Pearson chi-square/p-value		273.73/p-value < 0.001					

Associated factors with ED

The bivariate analysis demonstrated significant associations between several risk factors and ED status, highlighting the importance of these variables in the development or worsening of ED.

Age category was strongly associated with ED status (p-value < 0.001). The prevalence of ED increased with age, with the highest proportion of ED cases seen in the 51 to 60 years group (149, 73.4%) compared to younger age groups. The highest prevalence of ED was seen in patients with lower levels of education (primary education) (75, 65.8%), while individuals with tertiary education had the lowest prevalence (3 out of 10, 30.0%). This result was not statistically significant (p-value = 0.069).

Smoking was shown to have a negative association with ED (p-value = 0.002); however, 46.3% (37 out of 80) of smokers had ED, while 66.1% (144) of non-smokers had ED.

The presence of hypertension was significantly associated with ED (p-value = 0.026), with 64.4% (143) of hypertensive individuals having ED compared to only 50% (38) of non-hypertensive individuals. The duration of hypertension also showed a significant association with ED (p-value = 0.034), with longer hypertension duration (>10 years) associated with a higher prevalence of ED (51, 70.8%). Patients with diabetes had a high percentage of ED (119, 64.0%) compared to patients with no diabetes (62, 55.4%). This result was not statistically significant (p-value = 0.14). Further, patients who had diabetes for less than 10 years showed a prevalence of 61.6% (85 out of 138) ED; meanwhile, patients who had diabetes for more than 10 years showed a prevalence of 70.8% (34 out of 48) ED. This result is also statistically not significant with regression analysis (Exp B = 1.373; 95% CI: 0.978-1.928, p-value = 0.067). Other factors studied, such as high body mass index (BMI > 25 kg/m²), dyslipidemia, heart failure, alcohol use, and taking beta-blockers, did not show any significant association (p-value > 0.05) as shown in Table 2.

**Table 2:** Demographic and clinical data of the studied population with erectile dysfunction status ED: erectile dysfunction; DM: diabetes mellitus; HT: hypertension; BMI: body mass index; CAD: coronary artery disease; HF: heart failure; EF: ejection fraction; p: p-value A p-value less than 0.05 was considered significant; p-values meeting this criterion are bolded.

Variable	Category	No (%)	ED status		Statistics (Pearson chi-square/Fisher’s exact test and p-value)
			ED (181)	non-ED (117)	
Age	18 to 40 years	18 (6.0%)	2 (11.1%)	16 (88.9%)	47.55, p < 0.001
	41 to 50 years	77 (25.8%)	30 (39.0%)	47 (61.0%)	
	51 to 60 years	203 (68.1%)	149 (73.4%)	54 (26.6%)	
Education	Primary	114 (38.2%)	75 (65.8%)	39 (34.2%)	5.36, p = 0.069
	Secondary	174 (58.4%)	103 (59.2%)	71 (40.8%)	
	Tertiary	10 (3.4%)	3 (30.0%)	7 (70.0%)	
Smoking	Yes	80 (26.8%)	37 (46.2%)	43 (53.8%)	9.62, p = 0.002
	No	218 (73.2%)	144 (66.1%)	74 (33.9%)	
Alcohol user	Yes	109 (36.6%)	61 (56.0%)	48 (44.0%)	1.643, p = 0.2
	No	189 (63.4%)	120 (63.5%)	69 (36.5%)	
BMI (kg/m^2^)	>25 kg/m^2^	120 (40.3%)	71 (59.2%)	49 (40.8%)	0.21, p = 0.648
	≤25 kg/m^2^	178 (59.7%)	110 (61.8%)	68 (38.2%)	
DM	Yes	186 (62.4%)	119 (64.0%)	67 (36.0%)	2.17, p = 0.14
	No	112 (37.6%)	62 (55.4%)	50 (44.6%)	
DM duration	Nil	112 (37.6%)	62 (55.4%)	50 (44.6%)	3.45, p = 0.178
	≤10 years	138 (46.3%)	85 (61.6%)	53 (38.4%)	
	>10 years	48 (16.1%)	34 (70.8%)	14 (29.2%)	
HT	Yes	222 (74.5%)	143 (64.4%)	79 (35.6%)	4.93, p = 0.026
	No	76 (25.5%)	38 (50.0%)	38 (50.0%)	
HT duration	Nil	76 (25.5%)	38 (50.0%)	38 (50.0%)	6.774, p = 0.034
	≤10 years	150 (50.3%)	92 (61.3%)	58 (38.7%)	
	>10 years	72 (24.2%)	51 (70.8%)	21 (29.2%)	
Dyslipidemia	Yes	171 (57.4%)	101 (59.1%)	70 (40.9%)	0.471, p = 0.492
	No	127 (42.6%)	80 (63.0%)	47 (37.0%)	
CAD classification	CAD	217 (72.8%)	152 (70.0%)	65 (30.0%)	29.00, p < 0.001
	No CAD	81 (27.2%)	29 (35.8%)	52 (64.2%)	
HF (EF ≤ 40%)	Yes	11 (3.7%)	8 (72.7%)	3 (27.3%)	Exact Sig (2-sided) 0.537
	No	287 (96.3%)	173 (60.3%)	114 (39.7%)	
Taking beta blocker	Yes	161 (54.0%)	101 (62.7%)	60 (37.3%)	0.584, p = 0.445
	No	137 (46.0%)	80 (58.4%)	57 (41.6%)	

Further regression analysis showed that only age and the presence of CAD showed significant associations with ED status. Males aged 41-50 had 5.01 times greater risk of having ED (CI: 1.04-24.1) compared to those aged 18-40. Similarly, males aged 51-60 had 20.74 times greater risk of having ED (CI: 4.49-95.9) compared to the 18-40 age group. Diagnosed CAD was associated with a 3.92 times higher risk of ED (CI: 2.2-7.03) (Table 3).

**Table 3:** Final variables in the model - logistic regression Variable(s) entered on step 1: CAD-classification, HT, DM, education, age, HF (EF ≤ 40%). CAD: coronary artery disease; HT: hypertension; DM: diabetes mellitus; HF: heart failure; EF: ejection fraction; SE: standard error; df: degrees of freedom; Sig.: significance; Exp B: exponentiated B

Step 5*	Statistics on final step							95% CI for Exp B	
	Variables	B	SE	Wald	df	Sig.	Exp B	Lower	Upper
	CAD present	1.368	0.297	21.264	1	<0.001	3.928	2.196	7.027
	Age	-	-	34.166	2	<0.001	-	-	-
	Age 51-60	3.032	0.781	15.067	1	<0.001	20.742	4.487	95.893
	Age 41-50	1.612	0.801	4.051	1	0.044	5.014	1.043	24.098
	Constant	-3.009	0.798	14.212	1	<0.001	0.049	-	-

## Discussion

This study included 298 male patients undergoing coronary angiography, where the prevalence of ED was found to be 60.7% (181 out of 298 patients), and 70% (152 out of 217 patients) of these patients were also diagnosed with CAD. Notably, the prevalence of ED was significantly higher in patients with severe CAD, aligning with other findings where ED has been used as an early marker for cardiovascular issues.

The significant association between age and ED severity reflects the natural progression of both erectile and cardiovascular health with aging. Vascular aging, characterized by endothelial dysfunction and reduced arterial elasticity, is likely responsible for the increased prevalence of ED in older participants. This finding is consistent with prior studies that have established age as a critical risk factor for both cardiovascular disease and ED [11]. Education showed some association with ED status. Educational attainment influences lifestyle choices, but it does not show a direct correlation with ED severity. Regardless of education level, factors such as smoking and diet may play a more substantial role in ED outcomes [12].

Hypertension has a well-established relationship with ED due to its effects on endothelial dysfunction and arterial stiffness. The current study also demonstrated a significant positive association between HT and its duration with ED severity, suggesting that managing hypertension may be key to mitigating ED symptoms. This finding is in line with existing research that identifies hypertension as a key driver of vascular damage and ED [13].

Diabetes mellitus and its duration also showed no statistically significant association with ED; however, the role of chronic hyperglycemia in damaging the vasculature and exacerbating ED was emphasized in previous findings, which show how diabetes, through mechanisms like neuropathy and endothelial dysfunction, significantly increases the risk of severe ED [14]. The lack of significance may be due to sample size limitations, glycemic variability, or stronger confounding factors such as age and CAD. The findings suggest that comprehensive diabetes care remains essential for reducing ED risk and maintaining vascular integrity.

Heart failure with reduced ejection fraction is often associated with vascular and neuroendocrine imbalances affecting the ED. However, no significant association with ED was observed, possibly due to the small sample size (n = 11) and effective symptom management in these populations, which may reduce its impact on ED [15].

Although dyslipidemia is a known cardiovascular risk factor, its association with ED was not significant, likely due to the protective pleiotropic effects of statins commonly used by participants to manage dyslipidemia. Statins can enhance nitric oxide bioavailability, which is crucial for erectile function. These benefits could mask any potential negative impact of dyslipidemia on ED, resulting in a lack of significant correlation [16].

Inability to collect smoking status in pack-years or smoking intensity may not capture the true exposure, which could lead to a negative association between smoking and ED; however, smoking was still prevalent among patients with ED (46.2%). Furthermore, this finding may be attributable to lower smoking intensity or cumulative exposure among patients, resulting in attenuated vascular impairment. Furthermore, genetic resilience to endothelial dysfunction and compensatory lifestyle interventions, such as regular exercise or dietary adjustments, might diminish smoking’s deleterious effects. Additionally, the influence of smoking may be eclipsed by more potent determinants, such as hypertension or diabetes, within this study. Smoking has a direct impact on vascular health through endothelial damage and oxidative stress, correlating strongly with both ED prevalence and severity. Smoking cessation has been found to potentially improve erectile function over time due to the restoration of endothelial function [17].

While moderate alcohol use may have vasodilator effects, its relationship with ED remains complex. Our study found no significant association, potentially due to controlled levels of intake among participants or other overriding factors like hypertension and diabetes mellitus that were more directly linked to ED [18].

The lack of significant association between BMI over 25 kg/m² and ED may result from individual metabolic resilience, which can mitigate obesity's adverse vascular effects. Studies also indicate that lifestyle factors, such as physical activity, may counterbalance BMI's impact on endothelial function, reducing its influence on ED and CAD. Furthermore, the effect of obesity may be overshadowed by more impactful variables, such as hypertension and diabetes, which have stronger established links to ED and CAD severity [19].

Beta-blockers are known for side effects that can include sexual dysfunction, yet no significant relationship with ED was noted. This may be due to differences in individual drug responses or concurrent use of other medications that mitigate beta-blockers’ effects on erectile function [19].

This study examines the intricate relationship between ED and CAD, both of which reflect systemic vascular pathology characterized by endothelial dysfunction, atherosclerosis, and diminished nitric oxide bioavailability. These mechanisms impair arterial elasticity and compromise blood flow to the coronary and penile vasculature, thereby linking the pathogenesis of ED and CAD. Notably, ED often precedes symptomatic CAD, and the adjusted odds ratio with ED and CAD was 3.93 (CI: 2.20-7.03), strongly indicating its risks and positioning it as a potential early biomarker of cardiovascular compromise [20].

Limitations

The study reached 73.9% (298 out of 403) of the sample size and was confined to one major tertiary hospital, which may affect the generalizability of the findings; hence, the results may not accurately reflect the prevalence of ED in all patients with CAD. However, the Teaching Hospital is the only tertiary hospital in the region where the findings give a reflection of the whole population picture of the region. The inclusion criteria specify that only patients aged 18 to 60 are considered. This age restriction may exclude older patients who could also experience ED, potentially underestimating the true prevalence of the condition in the general population. The study employed a descriptive cross-sectional design, which captures data at a single point in time. This design limits the ability to establish causal relationships between ED and CAD, as it does not account for changes over time or the progression of either condition. The sensitive nature of ED may lead to underreporting or reluctance among patients to disclose their symptoms. This could result in an inaccurate assessment of the prevalence of ED among the study participants.

Furthermore, the inability to collect smoking status in pack years or smoking intensity may not capture the true exposure and could be a potential limitation in the analysis. The argument for routine ED screening in patients with CAD is supported by the strong pathophysiological and epidemiological overlap between the two conditions. However, we recognize that several confounding factors, including age, diabetes mellitus, hypertension, dyslipidemia, and medication use, may independently contribute to ED. Furthermore, due to the cross-sectional nature of this study, the possibility of reverse causation, whereby established CAD or its treatment may contribute to the development or worsening of ED, cannot be ruled out. These limitations highlight the need for longitudinal studies to better establish the directionality and causal nature of the ED-CAD relationship.

## Conclusions

Findings from this study confirm the strong association between ED and CAD, favoring the advocacy for routine ED assessments in patients with cardiovascular risk profiles, thus enhancing early intervention strategies and preventive care approaches. The IIEF scores could serve as adjunctive tools in detecting early CAD, particularly in high-risk groups such as diabetic and hypertensive patients with additional cardiovascular risk assessment. Given the strong association between ED severity and multi-vessel CAD, future prospective control studies should explore the potential benefits of using ED as a marker for initiating early cardiovascular interventions. Additionally, research into the role of phosphodiesterase-5 inhibitors, commonly used to treat ED, may provide insights into their protective cardiovascular effects. Screening of ED could provide guidance in selecting antihypertensive medications, as hypertension was found to be an important risk factor in the study.
